# Engineered Extracellular Vesicles: Tailored-Made Nanomaterials for Medical Applications

**DOI:** 10.3390/nano10091838

**Published:** 2020-09-15

**Authors:** Kenny Man, Mathieu Y. Brunet, Marie-Christine Jones, Sophie C. Cox

**Affiliations:** 1 School of Chemical Engineering, University of Birmingham, Edgbaston, Birmingham B15 2TT, UK; k.l.man@bham.ac.uk (K.M.); MYB925@student.bham.ac.uk (M.Y.B.); 2 School of Pharmacy, Institute of Clinical Sciences, University of Birmingham, Edgbaston, Birmingham B15 2TT, UK; m.c.jones@bham.ac.uk

**Keywords:** extracellular vesicles, exosomes, microvesicles, nanomedicine, regenerative medicine, EV engineering, artificial EVs, EV-functionalised biomaterials

## Abstract

Extracellular vesicles (EVs) are emerging as promising nanoscale therapeutics due to their intrinsic role as mediators of intercellular communication, regulating tissue development and homeostasis. The low immunogenicity and natural cell-targeting capabilities of EVs has led to extensive research investigating their potential as novel acellular tools for tissue regeneration or for the diagnosis of pathological conditions. However, the clinical use of EVs has been hindered by issues with yield and heterogeneity. From the modification of parental cells and naturally-derived vesicles to the development of artificial biomimetic nanoparticles or the functionalisation of biomaterials, a multitude of techniques have been employed to augment EVs therapeutic efficacy. This review will explore various engineering strategies that could promote EVs scalability and therapeutic effectiveness beyond their native utility. Herein, we highlight the current state-of-the-art EV-engineering techniques with discussion of opportunities and obstacles for each. This is synthesised into a guide for selecting a suitable strategy to maximise the potential efficacy of EVs as nanoscale therapeutics.

## 1. Introduction

There is a strong incentive to develop acellular technologies capable of circumventing the regulatory hurdles associated with cell-based therapies [1]. One such strategy relies on nanoparticle-mediated delivery of functional macromolecules and is a promising acellular approach for the treatment of various diseases. Specifically, in the last decade, the field of extracellular vesicles (EVs) has rapidly expanded as did our understanding of the roles these nanosized lipid particles play in various biological processes from the modulation of the immune system to bone regeneration [2]. This increasing body of evidence suggests that EVs have the potential to strongly influence the future direction of healthcare technologies. EVs are defined as phospholipid nanoparticles, released from pro- and eukaryotic cells that contain complex biological cargos, such as nucleic acids and proteins. The membrane of EVs is composed of a phospholipid bilayer containing receptors, major histocompatibility complex molecules and tetraspanins (CD9, CD63 and CD81), while their lumen may contain nucleic acids (mRNA, miRNA, non-coding RNAs and DNA), enzymes, proteins and other signalling molecules [3,4,5]. There is a growing body of literature concerning molecules that EVs may transfer, which includes: nucleic acids, enzymes, proteins, other signalling molecules and immunogenic materials. Given the focus of this review is engineering methods to augment the therapeutic potential of EVs, further information on the extensive cargo of these nanoparticles may be found elsewhere [5,6,7].

**Figure 1:**
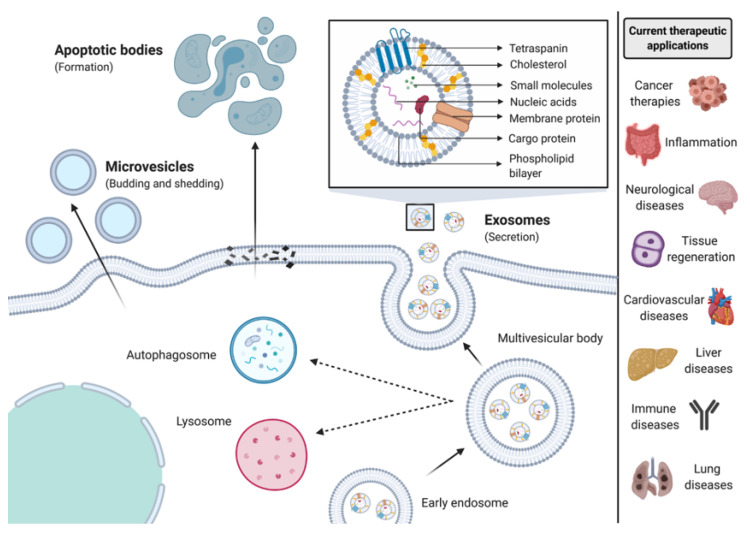
Biogenesis of extracellular vesicles (EVs) and potential applications of these nanoparticles in tissue engineering and regenerative medicine.

Three subtypes of EVs have been identified, which can be classified based on their biogenesis, composition and diameter (Figure 1). Exosomes are formed from the endosomal network and are released via the fusion of multivesicular bodies with the plasma membrane; their sizes range from 30–150 nm [8]. Microvesicles are formed from outward blebbing of the cell membrane with a 50–1000 nm size range [9]. Finally, apoptotic bodies are formed from the plasma membrane and are released during apoptosis; these bodies are heterogeneous with sizes in the range of 500–2000 nm [10]. The majority of research in this field has focus on small EVs, exosomes and microvesicles in the range of 30–300 nm in diameter. Although, our knowledge of the route of EV biogenesis has grown in recent years, is it still not yet fully understood. Guidelines have been developed by the International Society for Extracellular vesicles providing the scientific community with a way to promote rigorous and homogenous EV research advances [11].

As EVs are secreted by all cells, they can be isolated from various tissues as well as from bodily fluids, including plasma, urine, breast milk, saliva and amniotic fluid [12]. These naturally-derived nanoparticles fulfil their role as essential mediators of tissue homeostasis by, (i) specifically binding to surface receptors and initiating downstream signalling cascades within the cell, (ii) triggering endocytosis to promote their uptake by the target cells and (iii) fusing with the cell membrane, allowing the release of its cargo directly into the cytosol [13]. EVs are integral in critical physiological processes such as inflammation and bone regeneration, and they are also heavily implicated with pathological conditions, such as cancer [14]. This has resulted in two rapidly growing EVs research fields: (1) EVs are essentially a fingerprint of their parental cell and as such may be used as diagnostic markers of pathological disease since these nanoparticles may reflect the status of the tissue of interest in vivo, and (2) the therapeutic administration of EVs for the treatment of damaged/diseased tissues.

Many of the beneficial effects once accredited to cell-based therapies are now believed to be attributed in some degree to the paracrine cargo packaged within EVs [15,16]. This has accelerated research into the use of EV-based therapeutics due to several advantages that they possess compared to cell-based approaches. For instance, the nanoscale size of EVs in comparison to cells confers numerous benefits such as decreased vascular occlusion [17,18], macrophage phagocytosis [19], ease of administration and increased extravasation through tumour vasculature [19,20]. Additionally, the composition of EVs may enhance therapeutic benefits when compared to similarly sized delivery systems such as liposomes and synthetically-derived nanoparticles. For example, EVs exhibit high physiochemical stability [21], innate biocompatibility and the inherent ability to communicate with cells through their interaction [7]. Moreover, it has been reported that EVs exhibit tissue-specific tropism and cell-selective fusion [17,22,23,24], however, several studies have reported that the majority of EVs accumulate within the liver, spleen and lungs [25], where minor changes in targeting can be achieved by the use of different cells sources, functionalising EVs with targeting ligands or the site of administration [26]. Hence, there is great precedence to enhance the biodistribution and cell-targeting capabilities of EVs, ultimately improving their therapeutic efficacy.

Despite growing promise, limitations associated with the scalability of EV manufacturing, purity and their therapeutic potency have hindered the clinical translation of these naturally-derived nanoparticles [27]. As such, researchers have investigated several engineering strategies to address these challenges and promote the use of EVs as nanomedicines. In this review, we will outline current approaches that have been developed for this purpose including: (1) re-engineering of the parental cell, (2) modification of isolated EVs, (3) fabrication of artificial EVs and (4) EV-functionalised biomaterials.

## 2. Re-Engineering of the Parental Cell

**Figure 2:**
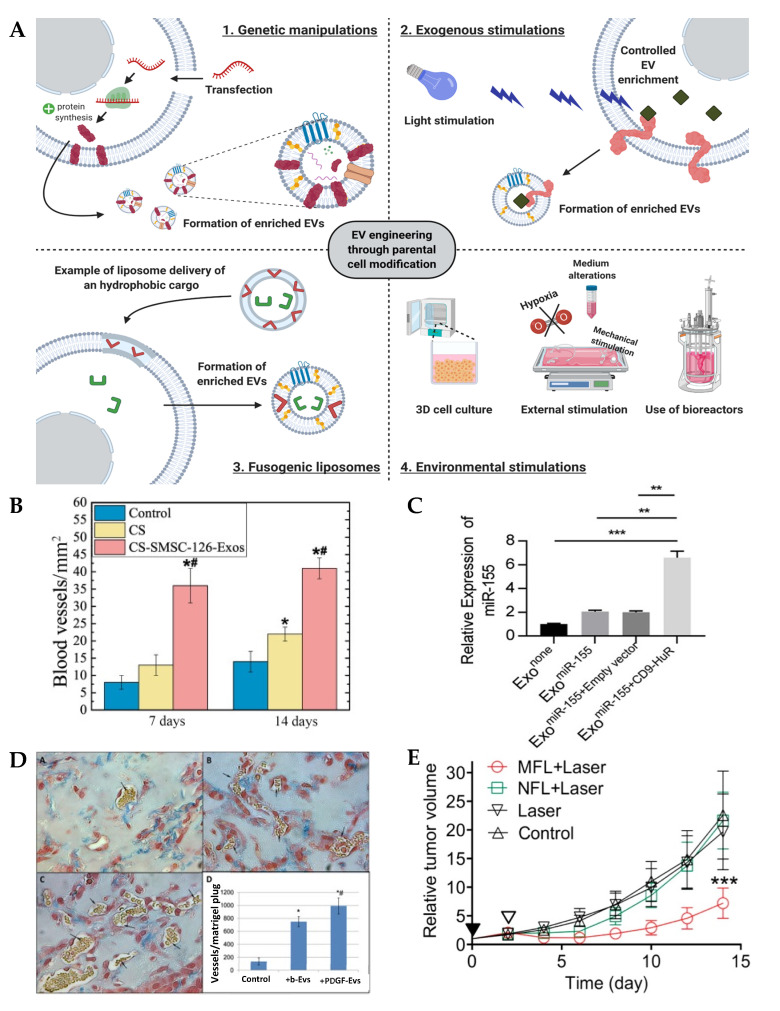
Engineering EVs by parental cell modification. (**A**) Schematic representation of methods to engineer EVs through parental cell genetic modification, stimulation with exogenous molecules, the use of fusogenic liposomes or environmental stimulation. (**B**) EVs derived from miR-126 overexpressing mesenchymal stem cells (MSCs) significantly increased vessel formation in a mice model. Reproduced from [31], with permission from John Wiley and Sons, 2016. *, *p* < 0.05 compared with control; #, *p* < 0.05 compared with CS. (**C**) CD9-human antigen R (HuR) enriched miR-155 into EVs with the miRNA efficiently delivered to the recipient cells, demonstrated by significantly increased miR-155 expression in the human monocytic cell line THP1. Reproduced from [34], with permission from American Chemical Society, 2019. **, *p* < 0.01; ***, *p* < 0.001. (**D**) Platelet-derived growth factor (PDGF)-stimulated EVs (+PDGF-EVs) exhibited substantially increased angiogenesis when compared to untreated EVs (+b-EVs). Black arrows indicate vessel formation. Adapted from [46], under the creative commons licence, 2014. *, *p* < 0.05 compared with control; #, *p* < 0.05 compared to b-Evs. (**E**) Reduced tumour volume observed in liposome-fused EVs (MFL + laser) when compared to liposomes treatment and laser irradiation alone. Reproduced from [51], with permission from American Chemical Society, 2015. ***, *p* < 0.001.

Re-engineering of the parental cell is achieved either through genome modification, stimulation with exogenous biomolecules, exposure to fusogenic liposomes or specific environmental factors with the aim of modulating de novo EV yield and therapeutic efficacy (Figure 2A) [28].

### 2.1. Genetic-Manipulation of Parental Cells

Modification of the parental cell genome allows the production of EVs enriched specifically with a desired therapeutic cargo. This method is thought to enhance the loading efficiency without compromising EV integrity or the functionality of the loaded compound [29,30]. The most common strategy for genome modification is via transduction, which triggers the production of a specific mRNA, non-coding RNA sequences (i.e., miRNA and siRNA) or protein within the parental cell. A few studies found this approach to be beneficial [31,32,33]. For example, overexpression of miR-126, a key mediator of angiogenesis, was triggered in synovium mesenchymal stem cells (MSCs) following transduction with miR-126-3p lentiviral vector. As a consequence of this treatment, MSCs secreted EVs that substantially enhanced angiogenesis in vitro, by promoting the migration and tube formation of human dermal microvascular endothelial cells, compared to vesicles derived from unmodified parental populations [31]. Moreover, when loaded into a chitosan hydrogel, the same EVs substantially increased vascularisation (>2-fold), in a diabetic rat model, compared to hydrogel alone (Figure 2B). In another study, transduction-triggered overexpression of C-X-C chemokine receptor 4 (CXCR4) in MSCs, and subsequent use of the harvested EVs, was found to promote angiogenesis in a rat M1 model (~2.3-fold increase compared to unmodified EVs), as well as preventing neonatal cardiomyocytes apoptosis in vitro (2.53-fold reduction compared to EV control) [32]. Although genome modification seems straightforward, there are several factors to consider, including the effect of the newly-integrated proteins on the viability and function of the parent cell, and the loading efficiency of the therapeutic molecule within the secreted EVs [33].

Due to issues regarding EV loading efficiency, several approaches have been investigated to further control the enrichment of the target molecules by harnessing the machinery involved in vesicle biogenesis. Li et al. engineered EVs for RNA loading by fusing the RNA binding protein human antigen R (HuR) with the tetraspanin membrane protein CD9. This successfully enriched miR-155 into EVs, with the functionally-intact miRNA efficiently delivered to recipient cells (Figure 2C) [34]. Moreover, several studies have identified other key miRNA sorting proteins that could be exploited to selectively enrich miRNAs in secreted EVs. For instance, Santangelo et al. identified the RNA binding protein synaptotagmin-binding cytoplasmic RNA-interacting protein (SYNCRIP) as an essential component to EVs miRNA sorting machinery within hepatocytes [35]. Similarly, Villarroya-Beltri et al. reported that sumoylated heterogeneous nuclear ribonucleoprotein A2B1 (hnRNPA2B1) controls the sorting of certain EVs miRNAs by binding to specific recognition motifs present in miRNAs [36].

Overall, the identification of proteins modulating EV content may provide a useful approach for the artificial, selective enrichment of clinically useful macromolecules within secreted EVs. Although this strategy has shown increased loading efficiency, a potential hurdle could be the ineffectual release of the tethered therapeutic molecule at the recipient cell [37]. Consequently, Yim et al. developed a system that allowed for the controllable, reversible loading and delivery of proteins into EVs. They utilised the photoreceptor cryptochrome 2 (CRY2), which interacted with CRY-interacting basic-helix-loop-helix 1 (CIB1) protein module fused to CD9. Irradiation with blue light resulted in the release of CRY2-conjugated cargo, thus providing a controllable release system for EVs cargo [38].

In addition to altering EV content, genetic modification of the parental cell has been investigated as a mean to modulate yield. Due to our increased understanding of the pathways involved in EV biogenesis, researchers have identified key components involved in their secretion. Several studies have reported the essential role of Rab proteins that regulate intracellular vesicle trafficking [39,40]. Similarly, plasma membrane GTPase, e.g., adenosine diphosphate ribosylation factor 6 (ARF6), is a key regulator of membrane remodelling, transport and vesicular trafficking. Laulagnier et al. overexpressed an effector of ARF6, phospholipase DR, resulting in a 2-fold increase in the number of exosomes released from cells, while its knockdown decreased exosome production 2-fold [41].

Although these studies demonstrate the promise of genetic modification, issues remain with regard to the transduction efficiency. Therefore, this approach is time-consuming and expensive compared to the direct modification of isolated EVs; also concerns over the safety of the process may complicate clinical translation [42,43]. Moreover the increased degree of manipulation suggests that EVs derived from genetically modified cells would likely be categorised as Advanced Therapy Medical Products (ATMP), hence would require more stringent safety testing when compared to EVs derived from non-genetically modified cells [44].

### 2.2. Exogenous Stimulation

Exogenous stimulation provides an alternative strategy in modifying EVs efficacy whilst overcoming limitations associated with genetic manipulation. This approach involves introducing a non-native bioactive molecule (growth factors, cytokines, drugs, etc.) into the cell culture medium, essentially appropriating the cellular biosynthesis machinery to manufacture and enrich EVs with the therapeutic molecule of interest. For example, MSCs cultured with erythropoietin (100 IU/mL) led to a 33% increased EV yield when compared to untreated cells, in addition to the vesicles being enriched with miRNAs, such as miR-299, miR-499, miR-302 and miRNA-200, leading to a more potent anti-apoptotic effect by exhibiting better restorative efficacy on TGF-β1-induced fibrosis in human renal proximal tubular epithelial cells in vitro and greater recovery in mice from renal injury of unilateral ureteral obstruction in vivo [45]. Similarly, it was demonstrated that platelet-derived growth factor (PDGF) (20 ng/mL) stimulated the secretion of EVs from adipose-derived MSCs (70% increase) and simultaneously enhanced the expression of pro-angiogenic factors (c-kit and SCF), while also inhibiting anti-angiogenic factors (angiostatin and endostatin) within the PDGF stimulated MSCs-EVs (Figure 2D) [46]. Qu et al. reported that the adenosine triphosphate activation of P2 × 7 membrane receptors triggers several cellular functions including membrane blebbing, sorting of endosomal contents and fusion with the multivesicular body to release the exosomes [47].

This strategy is, however dependent on the amount of the exogenous compound that is delivered to the cell, dictated by the strength of the compound–cell interaction [30]. Hence, biomolecules that exhibit weak binding capacity with cells, would rely on weak, non-specific interactions with the plasma membrane. Therefore, high compound concentration or longer exposure time would be necessary to enhanced loading efficacies, which could have a detrimental effect on cell viability [48]. Moreover, the detailed consequences of exogenous stimulation of parental cells are not yet fully understood. The expression of certain molecular species after stimulation might enrich EVs, potentially influencing their potency. Taken together, these studies indicate the considerable utility of exogenous stimulation of parental cells to enhance the therapeutic efficacy of secreted EVs.

### 2.3. Fusogenic Liposomes

Fusogenic liposomes are synthetic vesicles used as delivery vehicles to cells and they have been readily explored, due to the simplicity in functionalising and encapsulating both hydrophobic and hydrophilic molecules [49]. This approach has been adopted to introduce materials into the membrane and cytosol of the parental cell, in turn allowing for modification of the surface and cargo of secreted EVs [50]. Lee et al. used such an approach to fuse liposomes encapsulating the hydrophobic photosensitizer zinc phthalocyanine (ZnPc) into parental cells, resulting in the enrichment of the drug within the membrane of secreted membrane fusogenic liposomes (MFLs). This MFL enhanced therapeutic efficacy by reducing the relative CT26 tumour volume (~3.5-fold) compared to a non-fusogenic liposome (NFL) control (Figure 2E) [51]. The direct release of the liposome content into the cytosol is highly advantageous as it avoids the endocytotic pathway, therefore increasing the loading efficiency in EVs. For example, in Chinese hamster ovary cells, Kolašinac et al. showed that cationic fusogenic liposomes could reach a fusion efficiency ranging from 1 to 92% depending on the cationic lipid composition of the synthetic vesicles [52]. It is also illustrated that environmental conditions (ionic concentration, pH or buffer osmolality) impact the liposome fusion process which remains very low in physiological conditions [53]. Although this strategy has shown promise [54], lack of control over how the molecule is packaged into the vesicle and possible low entrapment of larger cargo may limit its wider applicability.

### 2.4. Environmental Stimulation

**Figure 3:**
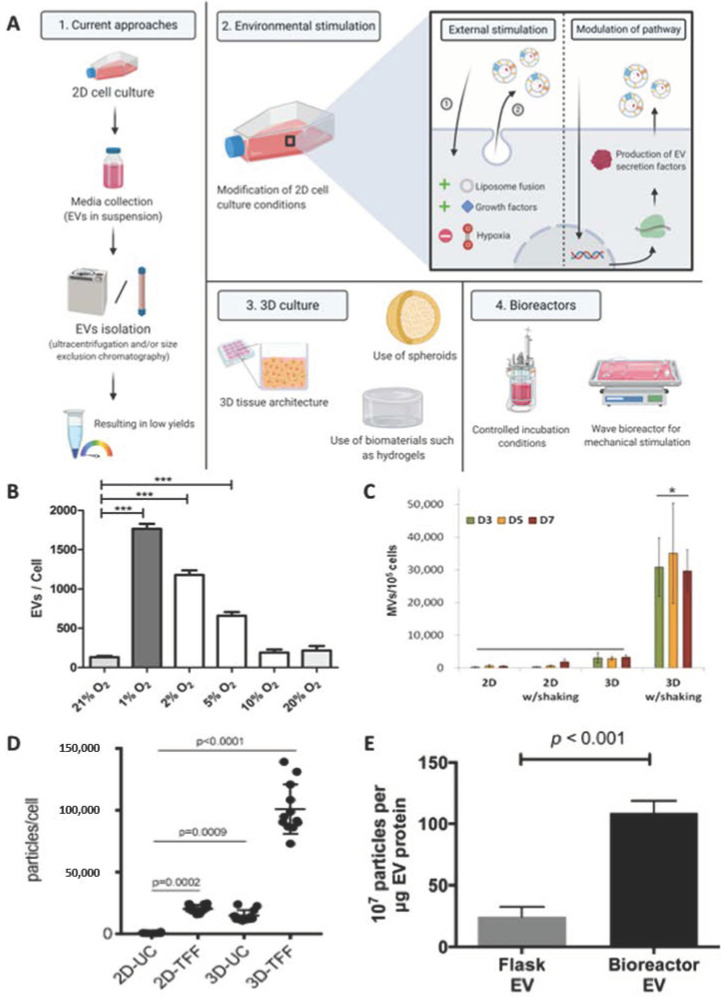
Methods of promoting EV scalability through environmental stimulation. (**A**) Schematic representation of strategies utilised to achieve scalability in EV manufacturing, such as the use of external stimulation, 3D culture platforms and bioreactor systems. (**B**) Hypoxic conditions enhanced the yield of EVs derived from endothelial cells compared to normoxic conditions. Reproduced with permission from [55], Elsevier, 2017. ***, *p* < 0.001. (**C**) 100-fold increase in EV quantity from MSCs cultured as spheroids with shaking during culture. Reproduced from [18], with permission from Springer Nature, 2018. *, *p* < 0.05. (**D**) Microcarrier-based 3D culture system combined with tangential flow filtration (3D-TIFF) enhanced MSCs yield 140-fold compared to cells culture in 2D conditions. Reproduced from [68], under the creative commons licence, 2018. (**E**) A hollow fibre bioreactor promoted EV yield 4-fold compared to 2D culture. Reproduced from [69], under the creative commons licence, 2016.

As research continues to foray into the utilisation of 3D culture environments, there also emerges the potential of harnessing environmental stimulation to scale-up EVs manufacturing for clinical applications. Several approaches have been explored to expand the synthesis of EVs by introducing external stimulation, modifying the culture platform and the use of bioreactors (Figure 3A).

#### 2.4.1. External Stimulation

Numerous approaches have been investigated to boost EVs production, such as altering the cells immediate environmental conditions. For instance, Burnley-Hall et al. induced hypoxia (1% O_2_) which increased EV synthesis 8-fold from human vascular endothelial cells when compared to normoxic conditions (21% O_2_), which was found to be mediated by the transcription factor hypoxia-inducible factor-1α (Figure 3B) [55]. Similarly, serum-deprivation increased EVs yield in human myeloma (2.5-fold) and neuroblastoma cells (10-fold) [56,57]. Eicholz et al. demonstrated that mechanical activation of osteocytes through the application of a physiological fluid shear during culture resulted in EVs exhibiting enhanced pro-osteogenic capacity [58]. A similar pro-osteogenic EV capacity was realised by Davies et al. when culturing parental osteoblasts in osteogenic media compared with a growth media control [59]. Though these studies demonstrate the potential of utilising external stimulation to increase EV yield, the alterations in culture conditions could modify the phenotype of the cells and consequently the compositions/therapeutic efficacy of their secreted products.

#### 2.4.2. 3D Culture Platforms

Due to the limitations that 2D culture systems infer on EV production, modification of the platform used to culture parental cells has been investigated to improve scalability and reproducibility. 3D culture systems have been favoured in recent years as a more representative model of in vivo conditions, including extracellular matrix composition, heterogeneous cell–cell interactions and 3D tissue architecture [60]. By conferring a more physiologically-relevant phenotype, this increased biomimicry is also more likely to result in the production of EVs with a more natural composition leading to a more physiological model to investigate the release of EVs for pathological conditions such as a cancer model or enhance their therapeutic potency for regenerative medicine.

Cha et al. cultured MSCs as spheroids within a microwell array system and reported a 100-fold increase in EV yield, when compared to cells grown on 2D surfaces, in addition to an increased therapeutic efficacy through the stimulation of angiogenic and neurogenic in vitro differentiation (Figure 3C) [18]. Moreover, the use of biomaterial constructs to replicate the cells’ physiological environment was shown to regulate EV paracrine signalling. MSCs cultured within a collagen scaffold exhibited a 2-fold increase in the secretion of EVs compared to cells grown on 2D tissue culture plastic (TCP). Moreover, these 3D cultured-derived EVs improved functional recovery of traumatic brain injury in a rat model, demonstrating both increased yield and therapeutic potency compared to TCP-derived EVs [61]. The use of 3D environments, however presents some limitations such as the difficulty in isolating EVs from non-porous biomaterials, including hydrogels [62]. Indeed, these systems may require further processing to extract EVs, which could damage their integrity and function. 3D constructs created from conventional scaffold fabrication techniques (e.g., solvent casting, gas foaming or phase separation) do not allow precise control of pore size, geometry and internal architecture [63], hence there are issues concerning the scalability and reproducibility of EV manufacture. However, the use of technologies such as 3D printing may enable increased reproducibility of 3D scaffolds for large-scale EV manufacturing [64].

#### 2.4.3. Bioreactors

Bioreactors have been extensively utilised for the large-scale synthesis of clinical-grade cells [65,66]. Recently, researchers have adapted this platform for the production of EVs. For example, Palviainen et al. reported that culturing prostate cancer cell lines within a two-chamber bioreactor system increased EV yield by >100 times and significantly altered the metabolite profiles when compared to culturing cells on TCP [67]. Similarly, Haraszti et al. reported a 140-fold increase in EVs derived from umbilical cord MSCs cultured in a microcarrier-based 3D culture system combined with tangential flow filtration when compared to EVs derived from 2D culture (Figure 3D) [68]. Utilising a hollow-fibre bioreactor, Watson et al. demonstrated a 4-fold increase in EV particle number when compared to conventional 2D culture (Figure 3E), which resulted in a 40-fold increased EV yield per volume of conditional medium compared to TCP [69]. These findings indicate the potential utility of bioreactor systems to scale-up EV manufacturing. However, further investigation is required to determine whether these systems alter the phenotype of the parent cell and composition of the resulting EVs. A summary of the methods discussed in this section to re-engineer the parental cell to enhance the therapeutic potency of EVs are shown in Table 1.

## 3. Modification of Isolated EVs

The strategy of bioengineering the parental cell predictably loads only a small proportion of the modified content into the secreted EVs, as the nanoparticle’s cargo represents a small portion of the parental cell cytoplasm, making this approach time- and cost-intensive. Direct modification of purified EVs may provide a more efficient therapeutic functionalisation strategy to enrich the surface membrane or cargo of these nanoparticles.

### 3.1. Passive Loading of EVs

#### EVs Incubation

Co-incubation is the simplest method to insert a cargo within EVs. Hydrophobic molecules can interact with the lipid membrane and diffuse into the aqueous cavity following the cargo concentration gradient [70]. Sun et al. successfully incorporated the anti-inflammatory agent, curcumin, within exosomes derived from EL-4 mouse lymphoma cell line (curcumin incubated with exosomes at 22 °C for 5 min) and reported enhanced drug stability in vitro and in vivo when compared to free curcumin in solution [71]. Moreover, Didiot et al. developed a highly efficient and reproducible method for loading hydrophobically modified small interfering RNAs (siRNAs) specific for Huntingtin mRNA within exosomes derived from U87 glioblastoma cells via co-incubation (siRNAs incubated with exosomes at 37 °C for 90 min). Exosomes loaded with siRNAs resulted in dose-dependent silencing of Huntington mRNA/protein in mouse primary cortical neurons [72]. Although this strategy has been successful, poor loading efficiency remains a major drawback. Additionally, this method is heavily dependent on the lipophilic properties of the cargo. Due to these issues, membrane permeabilisation strategies have been utilised for the active loading of therapeutic molecules into EVs.

### 3.2. Active Loading of EVs

#### 3.2.1. Electroporation

This method relies on the application of an electrical field that permeates the EV-membrane, allowing for the diffusion of a water-soluble load into the vesicle [73]. After removal of the field, membrane integrity is restored, preventing leakage of the EVs content. This strategy is particularly advantageous for the loading of large molecules when compared to other techniques. For example, it has been utilised to load siRNAs into EVs, with enhanced loading efficiencies compared to chemical transfection [74]. The main limitations are an increased risk of RNA precipitation/aggregation and disruption of EV integrity. Aggregation may result in an overestimation of the RNA and thus reduced efficacy [75].

#### 3.2.2. Sonication

EV loading via sonication utilises ultrasound, rather than electricity, to permeabilise the membrane [76,77]. Sonication is widely available and simple method to enrich EVs with therapeutic cargo during membrane reformation. However, similar to electroporation, applicability may be limited by the risk of permanent deformation of the EVs membrane and denaturation of the cargo [78]. Moreover, sonication may cause an increase in EV size [77,79], possibly compromising their inherent characteristics.

#### 3.2.3. Extrusion

This approach involves extruding a mixture of the EVs and the cargo of interest through a membrane with pore sizes ranging from 100–400 nm, at a set temperature [9]. Perforations in the EVs membrane are created, resulting in the influx of the cargo into the cavity. Extrusion may alter membrane composition due to constant membrane deformation. Notably, changes in the zeta potential of extruded vesicles have been observed [80].

#### 3.2.4. Freeze–Thawing

Loading EVs via freeze-thawing involves combining vesicles and the cargo at ambient temperature, freezing this mixture at −80 °C or in liquid nitrogen, then thawing back to ambient temperature; these cycles are repeated several times, ultimately allowing for the loading of the cargo through membrane deformation [81]. Although this method has resulted in medium loading efficiency when compared to other approaches, this procedure can induce EV aggregation [82]. Moreover, the freeze–thaw process may damage the functionality of the EVs as a result of mechanical disruption of the membrane. This method has been used to induce membrane fusion between EVs and liposomes, forming hybrid vesicles [71]. Specifically, Sato et al. co-incubated EVs derived from RAW 264.7 cells with fluorescently labelled liposomes and demonstrated that not only did fusion occur, but the cellular uptake of the hybrid EVs could be modified by modulating the lipid composition of the liposome [83].

#### 3.2.5. Saponin-Assisted Loading

The surfactant saponin can disrupt the EVs membrane via its interactions with cholesterol; this increases membrane permeability, leading to high loading efficiencies [79,80]. However, saponin concentrations need to be kept to a minimum to limit the risk of haemolysis and it is recommended to thoroughly wash EVs to remove trace surfactant [84].

Although these loading approaches have demonstrated an increased degree of cargo internalisation, the development of clinically relevant EVs is limited by issues concerning the robustness, reproducibility and scalability of currently available manufacturing methods [85]. Moreover, the permeabilisation procedures could potentially augment the native EVs structural integrity and targeting capabilities, whilst also impairing their immune-privileged status [70,86]. EV-loading strategies commonly utilised in the literature are summarised in Table 2.

### 3.3. Surface Modifications of EVs

The surface of EVs is integral in determining the biodistribution, cell-targeting and therapeutic capabilities of these naturally derived nanoparticles. Therefore, as with loading of therapeutic cargo in the EVs cavity, numerous strategies have been investigated to functionalise the surface including: hydrophobic insertion, synthetic lipid nanoparticle fusion and other covalent and non-covalent methods.

#### 3.3.1. Hydrophobic Insertion

The hydrophobic nature of the EVs membrane allows for the direct insertion of hydrophobic molecules on the nanoparticle’s surface. Endowing EVs with increased targeting capabilities has been extensively investigated for cancer therapeutics. For example, Huang et al. conjugated the aptamer AS1411 to cholesterol on the EVs membrane, which resulted in enhanced cell death (~2.8-fold compared to unmodified EVs) via increased delivery of vesicle-encapsulated miR-21 to leukaemia cells [87]. Similarly, Kim et al. functionalised the surface of macrophage-derived EVs containing the anticancer drug paclitaxel (PTX). This group incorporated aminoethylanisamide-polyethylene glycol onto the surface of EVs to target the overexpressed sigma receptors in lung cancer cells. These modified nanoparticles demonstrated increased accumulation in tumours following systemic administration within a Lewis lung cancer (LLC) mouse model, improving therapeutic outcomes [88]. Together these studies indicate that hydrophobic insertion provides a simple method for functionalising the surface composition of EVs.

#### 3.3.2. Synthetic Lipid Nanoparticle Fusion

The use of fusogenic liposomes has been investigated for the surface modification of isolated EVs. For instance, Piffoux et al. fused liposomes with EVs via a polyethylene glycol (PEG) mediated reaction [89]. The hybrid EVs exhibited increased delivery efficiency of loaded chemotherapeutic compounds when compared to drug-loaded liposomes and free drug. In an alternative approach, Kooijmans et al. developed nanobody-PEG-micelles by conjugating epidermal growth factor (EDGF) to phospholipid-PEG derivates [90]. The fusion of these micelles with EVs-derived from platelets or Neuro2A cells did not affect vesicle size distribution, morphology or protein composition. Moreover, increased specific binding to EDGF-overexpressing tumours was observed in addition to prolonged circulation following injection in Crl: NU-Foxn1^Nu^ mice bearing A431 human squamous carcinoma cell xenografts. Utilising this approach may augment the natural composition of the vesicle surface, which is integral to many of its homing capabilities and cellular interactions.

#### 3.3.3. Covalent Modifications

Click-chemistry is an example of covalent modification successfully utilised to functionalise the surface of EVs. The click reaction occurs when an azide chemical group reacts with an alkyne group forming a triazole bond [28]. This reaction offers selectivity with the conjugation site compared to other cross-linking methods, whilst also occurring under ambient conditions, thus avoiding damage to EV integrity and aggregation. Lee et al. inserted azide-lipids into the plasma membrane of EVs via membrane fusogenic liposomes and conjugated targeting peptides using click-chemistry to enhance cancer cell targeting [50]. In a similar approach, Smyth et al. successfully functionalised EVs derived from 4T1 breast cancer cells with 4-pentynoic acid in the presence of N-(3-(dimethylamino-propyl)-N′-ethylcarbodiimide and amine-reactive n-hydroxysuccinimide [91]. The alkyne-functionalised EVs were cross-linked azide-fluor 545 via copper-mediated click chemistry. However, it is important to note the potential cytotoxicity of the copper catalyst, which has motivated investigations of copper-free click approaches. Tian et al. conjugated alkyne-functionalised EVs with cyclo (Arg-Gly-Asp-D-Tyr-Lys) peptide [92]. Excitingly, these engineered EVs were shown to target lesions within ischemic brain tissue following intravenous administration and to successfully suppress inflammation and apoptosis at the injury site. Another interesting way to covalently modify the surface of EVs is via the conjugation of the lipids which represent the vast majority of the membrane composition. Lipid conjugation can be performed directly on EVs or as a parental cell modification as described in Section 2.2. Wan et al. grafted lipidated ligands to dendritic cell-derived nanovesicles loaded with PTX to actively target cancer cells [93]. These surface modified EVs exhibited a 6-fold and 3-fold treatment efficacy in vitro and in vivo compared to unmodified PTX-loaded nanovesicles. For RNA delivery, the conjugation of siRNAs to cholesterol led to an increased loading efficiency demonstrating an exponential correlation between the hydrophobicity of the siRNA lipid conjugate and its EV-loading efficiency [94].

Notably for these approaches, given the altered surface composition, it is important to explore any effects this functionalisation may have on therapeutic efficacy of the modified EVs.

#### 3.3.4. Non-Covalent Modifications

Non-covalent approaches to functionalise the EV membrane, such as receptor–ligand binding and electrostatic interactions, have also been reported in the literature [30]. Qi et al. conjugated superparamagnetic nanoparticles to the transferrin receptors of blood-derived EVs, which enhanced the targeting capabilities for cancer therapeutics [95]. Utilising a similar strategy, Maguire et al. attached biotinylated magnetic nanoparticles to transgenic biotin-acceptor peptides, introduced to EVs following the transfection of parental cells [96]. The challenge with this approach is the reproducible presentation of a functional ligand following the introduction of the transgene to the parental cell. However, the increased specificity of this strategy could provide numerous opportunities for the use of EVs for both pathological and regenerative processes.

Electrostatic interactions rely on the interaction of exogenous cationic species with the negatively charged groups on the EV membrane. For example, Nakase and Futaki utilised such an approach to attach cationic lipids and a pH-sensitive fusogenic peptide (GALA) to the surface of HeLa-derived EVs, producing positively-charged surfaces, which enhanced cell attachment and subsequent uptake [97]. It is however important to highlight that cationic molecules may cause cytotoxicity through cell membrane permeabilisation or thinning [98,99]. Moreover, typically cationic materials enter the cell by endocytosis, which leads to lysosomal degradation, hence resulting in poor EV loading efficacies [50]. Therefore, it is important to balance the increased uptake provided by a positive charge, against the risk of toxicity and premature lysosomal degradation.

Despite the progress made in EV surface engineering, realising efficient nanoparticle modification is not trivial. It is important to strictly control reaction conditions, such as temperature or pH, to avoid disruption to EV integrity and aggregation [30,82]. Nonetheless, these studies demonstrate the wide-ranging engineering approaches to enhance the biodistribution, cell-targeting and therapeutic capabilities of these naturally-derived nanoparticles. A summary of the methods discussed in this section to modify the surface composition of EVs are shown in Table 3.

## 4. Fabrication of Artificial EVs

The development of artificial EVs presents an opportunity to overcome the potential safety issues associated with those derived from allogenic sources. In addition, this approach circumvents the low procurement and extensive time associated with acquiring autologous EVs. The manufacturing of such systems is already well studied, with two approaches dominating the literature, (1) cell-derived nanovesicles (CDNs) and (2) EV-inspired liposomes (EVLs). Notably, use of microfluidics to increase manufacturing scale for either method is a rapidly growing research area.

### 4.1. Cell-Derived Nanovesicles (CDNs)

**Figure 4:**
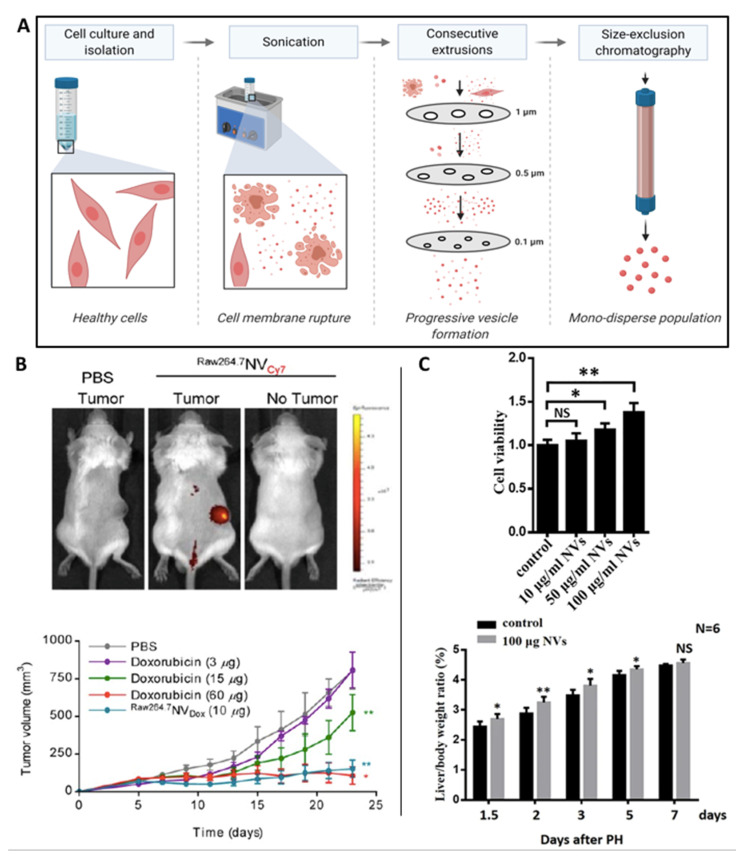
The use of cell-derived nanovesicles (CDNs) as an EV-mimetic system. (**A**) Schematic illustration of the process to fabricate CDNs from whole cells. (**B**) Macrophage-derived CDNs loaded with doxorubicin traffic to tumour tissue (upper panel) and reduced tumour growth compared to free-doxorubicin (lower panel). Reproduced from [102], with permission from American Chemical Society, 2013. *, *p* < 0.05; **, *p* < 0.01 compared to PBS. (**C**) CDN-derived from hepatocytes stimulated proliferation in vitro (upper panel) and liver regeneration in vivo (lower panel). Reproduced from [106], under the creative commons licence, 2018. NS, not significant; *, *p* < 0.05; **, *p* < 0.01.

CDNs represent a new class of bioinspired drug delivery systems, overcoming the extensive purification steps and low procurement yields of current EV-manufacturing methods [100]. Whole cells are disassembled by physical manipulation such as sonication and/or mechanical extrusion through serial filters of diminishing pore sizes (1, 0.5 and 0.1 μm) is typically used to create nanosized vesicle-like particles (50–200 nm) (Figure 4A). Starting from an equivalent number of cells, this method yields significantly greater quantity of nanoparticles compared to conventional EV-procurement methods, which are time and cost intensive [101,102,103]. Alternatives methods for CDNs production are also emerging, including microfluidic fabrication forcing cells through hydrophilic microchannels to generate CDNs which appears to be particularly suited as RNA or drug delivery systems (biomimetic approach) [104]. As CDNs are formed from whole cells, they exhibit a similar lipid and protein composition compared to the plasma membrane and therefore, do not require further surface functionalisation. Moreover, CDNs are advantageous compared to synthetic drug delivery systems as they exhibit prolonged circulation times, reduced clearance rate and lower risk of eliciting systemic toxicity without requiring additional engineering strategies [9]. Several studies have also demonstrated that CDNs are capable of shuttling endogenous cargo, such as RNAs and proteins to recipient cells similarly to native EVs.

It has been reported that during plasma membrane self-assembly, CDNs can encapsulate and deliver exogenous therapeutic molecules to targets cells. This suggests a role for these vesicles as viable drug carriers for the treatment of various diseases [105]. The nanoscale size of CDNs may be advantageous for cancer therapeutics due to their passive targeting via enhanced permeability and retention effect on the leaky vasculature within tumour tissues. Jang et al. utilised CDNs-derived from macrophages and monocytes, as drug carriers to successfully load chemotherapeutic agents into CDNs. The doxorubicin-loaded CDNs trafficked to tumour tissues, resulting in reduced growth compared to free doxorubicin administered intravenously to mice bearing CT26-tumor xenografts (Figure 4B) [102].

Successful utilisation of CDNs has also been demonstrated in the field of regenerative medicine. Wu et al. reported that CDNs derived from hepatocytes stimulated hepatocyte proliferation in vitro and liver regeneration in vivo by enriching target cells with sphingosine kinase 2 (Figure 4C) [106]. Han et al. demonstrated the fabrication of CDNs derived from MSCs for skin wound regeneration. It was reported that the CDNs exhibited 300 times increased productivity when compared to MSCs EVs. Moreover, these CDNs enhanced the proliferation and migration of primary fibroblast in vitro compared to MSC EVs and enhanced wound healing within a mouse model [104]. These studies demonstrate the potential utility CDNs may provide as an alternative to the use of EVs, however, it is envisaged that it would be difficult to control the final CDNs composition, which may impair manufacturing reproducibility and therapeutic efficacy.

### 4.2. EV-Inspired Liposomes (EVLs)

**Figure 5:**
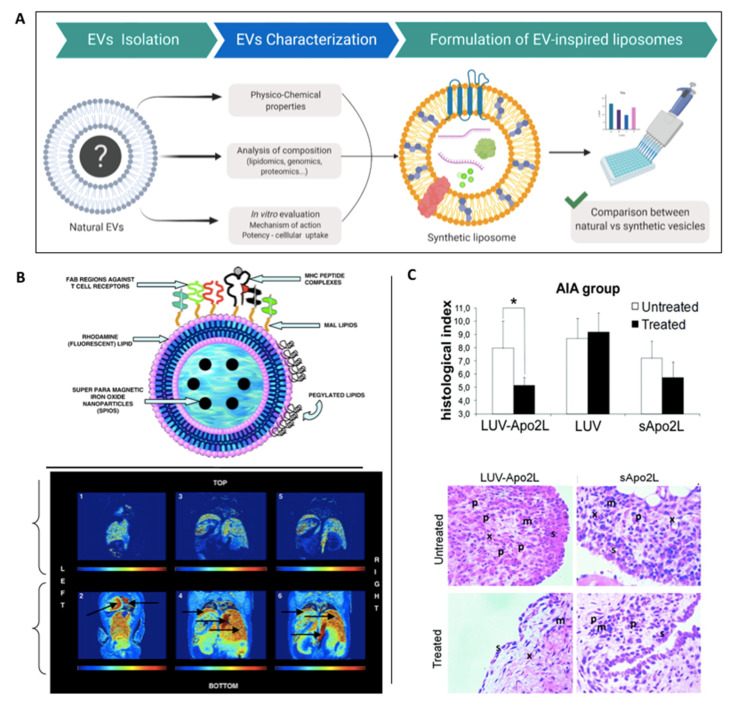
The use of EV-inspired liposomes as therapeutic nanoparticles. (**A**) Schematic illustration representing the general strategy in mimicking the composition and therapeutic potency of naturally-derived EVs with EV-inspired liposomes (EVLs). (**B**) Liposomes incorporated with MHC Class I peptide complexes and ligands to facilitate adhesion, early/late activation and survival T cell receptors (upper panel). Incorporation of superparamagnetic particles allows for the tracking of these EVLs in vivo (lower panel). Reproduced from [110], with permission from Elsevier, 2009. (**C**) Liposome conjugated with APO2L/TRAIL (LUV-APO2L) substantially reduced inflammation within a rabbit model of antigen-induced arthritis model. Reproduced from [112], with permission from John Wiley and Sons, 2010. *, *p* < 0.05.

Biomimicry of naturally-derived EV composition will likely result in replicating the therapeutic effects of these nanoparticles while overcoming issues regarding native EV purity and scalability. To facilitate this approach, it is vital to define and characterise the key therapeutic components within naturally-derived EVs (i.e., RNA, proteins, lipid and metabolites) (Figure 5A). Following this, EVLs can be fabricated utilising the minimum required elements to direct the desired therapeutic outcome, this does however assume that not all vesicular components are important for its native function [107]. One of the major advantages that EVLs have over the previously described strategies is the production of a distinct and pure population of nanoscale particles. This in combination with the ability to precisely control composition and scale production [108], highlights the considerable potential of liposome technology.

Although the use of EVLs is still in its infancy, a growing number of studies have demonstrated the potential of this approach. For example, Lu et al. fabricated EVLs (1,2-dioleoyl-sn-glycero-3-phosphocholine (DOPC)/Sphingomyelin (SM)/Cholesterol (Chol)/1,2-dioleoyl-sn-glycero-3-phospho-L-serine (DOPS)/1,2-dioleoyl-sn-glycero-3-phosphoethanolamine (DOPE)) (21/17.5/30 /14/17.5, mol/mol ratio)) to deliver an anti-VEGF siRNA to A549 cancer cells [109]. Compared to commercially available lipid-based transfecting agents (e.g., Lipofectamine 2000 and 1,2-dioleoyl-3-trimethylammonium-propane (DOTAP)), the EVLs displayed reduced cytotoxicity and increased storage stability over 30 days. Moreover, the EVLs exhibited substantially higher cell uptake (>2.73) and silencing potency (>3-fold) when compared to DOPC/Chol liposomes, however, displayed reduced delivery efficiency of oligonucleotides when compared to Lipofectamine 2000, DOTAP and other cationic lipids.

De La Pena et al. developed artificial vesicles mimicking the composition of dendritic cell-derived EVs, which are capable of directing T cell activation and modulating the immune response to primary, metastatic and relapsed tumours (Figure 5B). Liposomes were fabricated to contain major histocompatibility complex (MHC) Class I peptide complexes and ligands in their membrane to facilitate adhesion, early and late activation as well as survival T cell receptors. It was demonstrated that these artificial-EVs successfully activated and expanded antigen-specific T cells and were made traceable both in vitro and in vivo via magnetic resonance imaging as a result of superparamagnetic particle encapsulation [110]. Similarly, Martinez-Lostao et al. synthesised liposomes conjugated with Apo2 ligand/TNF-related apoptosis-inducing ligand (APO2L/TRAIL), as this group observed that T lymphocytes present in the synovial fluid of rheumatoid arthritis patients are sensitive to activation of both these molecules [111]. Within a rabbit model of antigen-induced arthritis, administration of these liposomes resulted in a more effective treatment when compared to soluble and unconjugated APO2L/TRAIL, with reduced inflammation and synovial hyperplasia observed in the knee (Figure 5C) [112]. Moreover, these liposome-bound APO2L/TRAIL-induced apoptosis on haematological tumour cell lines via caspase-3 activation, whilst also overcoming the chemo resistance of soluble APO2L/TRAIL [113].

Challenges presented with the use of EVLs, is to determine the influence of artificial surface compositions on the biodistribution and cell-targeting capabilities when compared to native-EVs [28]. Overall, these studies demonstrate the potential utility of liposome technologies in fabricating biomimetic EVs.

## 5. EV-Functionalised Biomaterials

**Figure 6:**
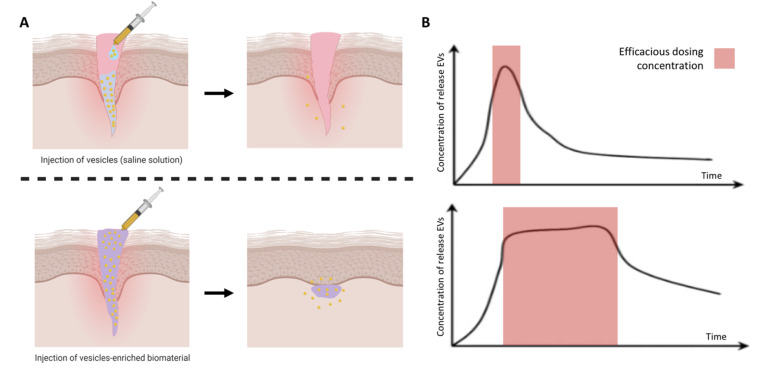
Therapeutic administration of EVs to the site of injury. (**A**) Schematic representation of the local delivery of EVs within an injectable biomaterial resulting in increased retention and therapeutic efficacy compared to delivery in solution. (**B**) Release kinetics of EVs from different delivery strategies. Burst release of EVs delivered in solutions, while sustained delivery observed from EV-encapsulated biomaterial systems. Adapted from [118], under the creative commons licence, 2018.

Researchers have investigated the therapeutic efficacy of EVs in vivo via intravenous delivery into the systemic circulation. Studies have reported systemically injected EVs are rapidly taken up by macrophages in the reticuloendothelial system and cleared from the body [114]. Hence, there is great necessity to increase the circulation half-life of EVs and promote their accumulation at the target site. Moreover, difficulties in manufacturing EVs in large quantities and high purity further emphasises the importance of increasing EVs half-life, potentially facilitating clinical translation [27]. In an attempt to solve these issues, research has moved towards local delivery of EVs, with greater therapeutic efficacy observed when compared to systemic administration. However, rapid EV clearance from the defect site is still observed and, in some cases, frequent dosing is required (Figure 6A) [115]. Biomaterials have been extensively utilised to aid the retention of cells and growth factors to overcome rapid clearance and achieve local activity. Therefore, to realise the potential of EVs as new regenerative tools, the use of biomaterials may provide a platform to enhance their bioavailability, sustain release and maximise regenerative capacity (Figure 6B) [116,117].

### 5.1. Considerations for Biomaterials Design

**Figure 7:**
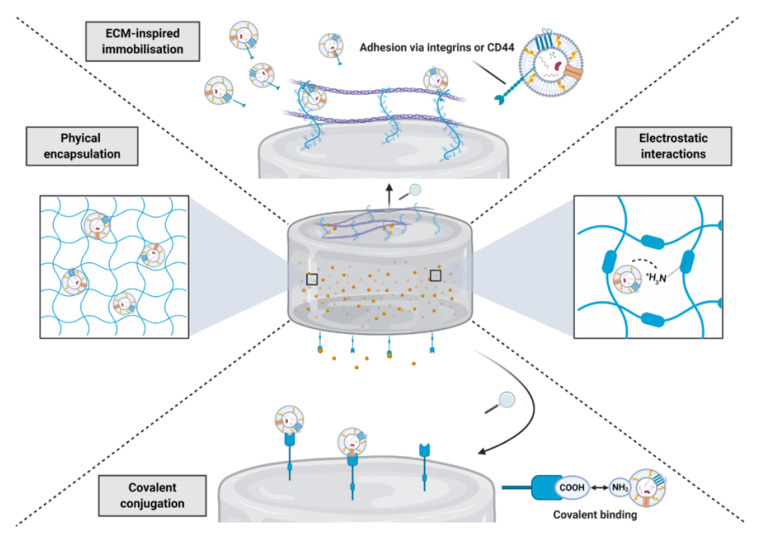
Strategies to immobilise EVs within biomaterials. Methods of EVs incorporation within biomaterials include physical entrapment, extracellular matrix (ECM)-immobilisation, electrostatic interactions and covalent conjugation.

The ideal biomaterial for administering EVs should control the release kinetics of these nanoparticles to sufficiently recruit endogenous cells of interest. It should also provide a suitable mechanical environment to support the defect site and a degradation rate that matches de novo tissue growth. Biomaterials are generally derived from natural or synthetic sources. Natural biomaterials provide a biomimetic environment and specific sites for cellular attachment (e.g., the Arg-Gly-Asp peptide sequence that can be modified to alginate), however, they are often associated with biological variability and limited mechanical strength [119,120]. Synthetic biomaterials (i.e., poly(lactide-co-glycolide) and poly(caprolactone)) are highly tuneable in terms of their architecture, mechanical strength and degradation rate, and overcome limitations regarding cost, supply and batch-to-batch variation associated with natural materials [121]. Composite biomaterials that harness the advantages of both systems have been increasingly utilised for the delivery of growth factors. The class of biomaterials most utilised in the literature to deliver EVs are hydrogels due to the ease of incorporation, tuneable mechanical properties and the possibility for administration by injection. Numerous approaches have been trialled to tune the release kinetics of growth factors, including EVs depending on the clinical application (Figure 7).

Controlling the porosity of the biomaterial, along with its degradation rate, will ultimately affect the EVs release rate and favouring nanosized-pores can help prevent the bulk release observed following local delivery [121]. However, cellular and vascular infiltration within the biomaterial may be compromised if pores are too small and, in this instance, macroporosity may be desirable. Natural biomaterials exhibit increased binding to growth factors due to the biomimicry of the extracellular matrix (ECM). Moreover, it has been reported that EVs can bind to ECM components, such as collagen and hyaluronan via integrins and CD44 respectively, indicating natural biomaterials may be superior for EVs retention [122]. With EVs exhibiting a negative charge, this presents the potential to immobilise these nanoparticles within biomaterials via electrostatic interactions [123]. Conjugating EVs with the biomaterial via covalent modification would provide the strongest immobilisation method, which would eliminate burst release and prolong delivery, similar to what has been shown for growth factors [124,125]. In addition to controlling the EVs release kinetics, the mode of delivery is an essential consideration for biomaterial design. Injectable delivery of hydrogels is highly advantageous as it allows for minimally invasive administration to the defect site, although if the defect requires substantial structural support, surgical implantation of the biomaterial may be appropriate. Numerous studies have delivered EVs within biomaterials for the regeneration of various tissues, highlighted in Table 4.

**Figure 8:**
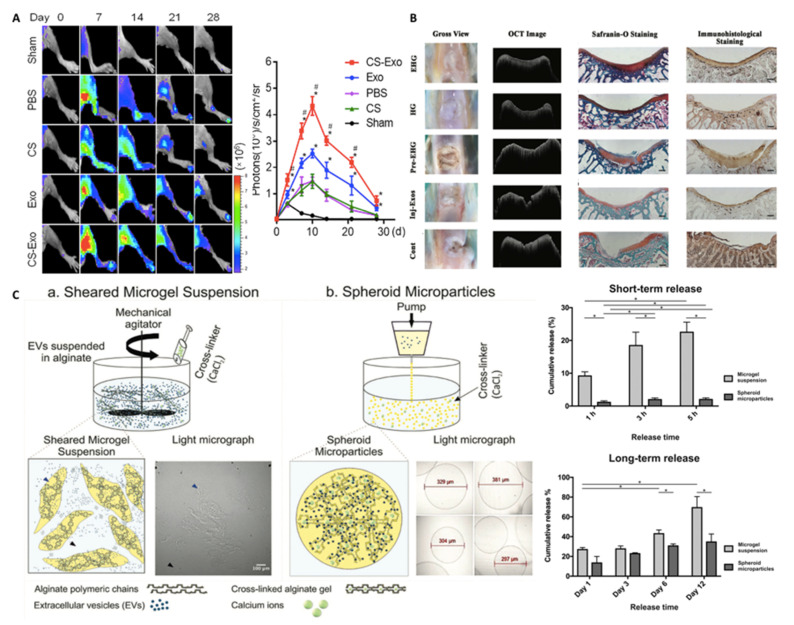
Encapsulation of EVs within different hydrogel systems. (**A**) MSC-derived exosomes with an injectable hydrogel for hindlimb ischemia treatment. EVs encapsulated within chitosan hydrogel demonstrated enhanced retention at the site of injury when compared to local delivery with EVs alone. Adapted from [134], with permission from American Chemical Society, 2018. *, *p* < 0.05 compared to PBS; #, *p* < 0.05 compared to Exo. (**B**) Superior reparation of articular full-thickness rabbit defects treated with in situ forming EV-functionalised hydrogel (EHG) when compared to hydrogel alone (HG), pre-formed EV-hydrogel (Pre-EHG), EV delivery alone (Inj-Exos). Adapted from [136], with permission from Royal Society of Chemistry, 2017. (**C**) Development of two technologies capable of controlling the release kinetics of encapsulated EVs. Reproduced from [137], with permission from John Wiley and Sons, 2019. *, *p* < 0.05.

Zhang et al. investigated delivering human placental MSC-derived EVs embedded within a chitosan hydrogel for muscle regeneration [134], which was found to increase retention at the defect compared to EVs injection in solution (Figure 8A). Sustained release at the defect site was observed up to 28 days, with improved functionality evidenced by increased capillary formation and tissue regeneration. In a similar study, Chen et al. evaluated the regenerative capacity of endothelial progenitor cell-derived EVs following myocardial infarction [126]. EVs were incorporated within a composite hyaluronic acid gel that exhibited shear-thinning behaviour and sustained EVs release over 21 days. Excitingly, delivery of EVs within this hydrogel restored the functionality of the damaged myocardium to the same extent as a cell-based approach within a rat myocardial infarction model. Biomaterial functionalisation with EVs has also demonstrated potential for hard tissue engineering applications [131,135]. For instance, Liu et al. studied the impact of encapsulating human induced pluripotent stem cell-derived exosomes within a hydrogel glue (*o*-nitrobenzyl alcohol moieties-modified hyaluronic acid and gelatin) for articular cartilage regeneration [136]. They demonstrated that the EV-functionalised hydrogel promoted chondrocyte proliferation in vitro and substantially enhanced articular cartilage repair in a rabbit defect model when compared to EVs delivered in solution (Figure 8B).

Although numerous studies have demonstrated the efficacy of EV retention within biomaterials for tissue regeneration, there are a lack of systems that offer modifiable release kinetics that could be tailored to a specific clinical application. Nikravesh et al. developed two technologies for controlling the structure of alginate-based microgel suspensions, which demonstrated augmented EV release kinetics [137]. Microparticles formed using a shearing technique were compared to those manufactured using vibrational technology, resulting in either anisotropic sheet-like or spheroid particles, respectively (Figure 8C). A significantly greater number of EVs were released from the suspensions formed by shearing (2-fold) compared to the spheroids. Ultimately, alterations to the physical structure of the hydrogel were shown to tailor EV release, while providing ideal material characteristics for clinical injection. Overall, these studies highlight the tremendous progress made in promoting the therapeutic efficacy of EVs through biomaterial functionalisation.

### 5.2. Challenges and Future Perspective

In recent years, EVs have emerged as essential regulators of normal physiological processes and pathological conditions, and present exciting opportunities for development as novel acellular tools for regenerative medicine and as diagnostic markers [17,138]. However, the potential utility of EVs has been limited due to issues regarding their scalability and therapeutic efficacy. Therefore, numerous strategies have been investigated to enhance the potency of these nanoparticles beyond their native function. From reviewing the current state of the literature, several challenges need to be overcome to help support the onward clinical progression of these nanoparticles. This includes procuring EVs from physiologically relevant environments, the scalability of manufacturing, the development of artificial nanoparticles and controlling EVs in vivo release kinetics.

Understanding the biology of EVs by analysing their composition is important for the translation of any cell modification techniques of these nanoparticles. However, the majority of studies elucidating EVs composition and hence their mechanism of action, isolate these nanoparticles from 2D TCP, a highly artificial system compared to the cells’ native microenvironment [139]. For example, Thippabhotla et al. demonstrated that EVs derived from cervical cancer cells cultured on TCP displayed a significantly altered RNA and DNA content when compared to that of EVs derived from 3D models, with the later exhibiting ~96% RNA similarity to in vivo derived EVs (compared to ~80% similarity of TCP derived EVs) [140]. Improving the biomimicry of EVs isolated from in vitro systems allows for more accurate native replication using various engineering strategies utilised. In this regard, harnessing bio fabrication methods such as 3D printing would allow for the large-scale fabrication of 3D constructs with precise control in the scaffold’s architecture, which conventional fabrication techniques lack [60,63]. In turn, harnessing these layer-by-layer technologies would lead to scalable and reproducible manufacture of more physiologically relevant EVs [64].

In addition to the production of more physiologically relevant EVs, the large-scale manufacture of therapeutically viable EVs is essential to meet clinical demand [28]. Several studies have reported the use of bioreactor systems to achieve EVs scalability, with significantly increased quantity derived from the use of these systems [141,142]. However, at present, there is limited knowledge of how these bioreactor systems affects the composition of EVs and their therapeutic potency. Moreover, it is a particularly challenging task to procure EVs from primary cells on a large-scale since they have a limited passage window [10]. In addition, the use of bioreactors systems may aid in fabrication of clinical grade EVs. Several studies have reported the development of good manufacturing practice-grade standard protocols for the isolation of EVs from a variety of cell source including MSCs working towards the large-scale production of good manufacturing practice (GMP)-compliant productions [143,144]. Therefore, future directions should focus on investigating the effects of bioreactor parameters and systems on EVs production [62].

The clinical translation of EVs has been limited due to issues associated with manufacturing scalability, purity and therapeutic potency [27]. This has accelerated research in the development of modified or fully artificial EVs. Although the development of artificial EVs is still in its infancy, the majority of studies have focused on encapsulating simple molecules (i.e., RNA or drugs and magnetic or fluorescent probes) [109,110], utilising well-established cell manipulation techniques. It is much more difficult to incorporate proteins within the EV membrane in the correct orientation whilst maintaining their tertiary structure, which is key to their functional activity [30]. Hence, technologies that are capable of imparting enhanced functionality via the incorporation of these transmembrane proteins would allow for increased biomimicry and ultimately improve their utility as smart nanoscale therapeutics. Moreover, the altered composition and integrity from modified natural EVs or artificial nanoparticles will impact their biodistribution, cell targeting and preservation capabilities [28]. Consequently, it is pertinent to determine whether the engineering processes affect the EVs therapeutic efficacy and long-term storage stability [27].

Finally, the growing use of biomaterials to control the delivery and retention of EVs for different clinical needs have shown promise in the literature [126,131,133]. However, there are still several issues obstructing the clinical translation of EV-functionalised biomaterials. These include optimising the dosing regimen to maximise therapeutic response in vivo, controlling the release kinetics at the site of injury and the role the biomaterial plays in supporting tissue regeneration [121].

**Figure 9:**
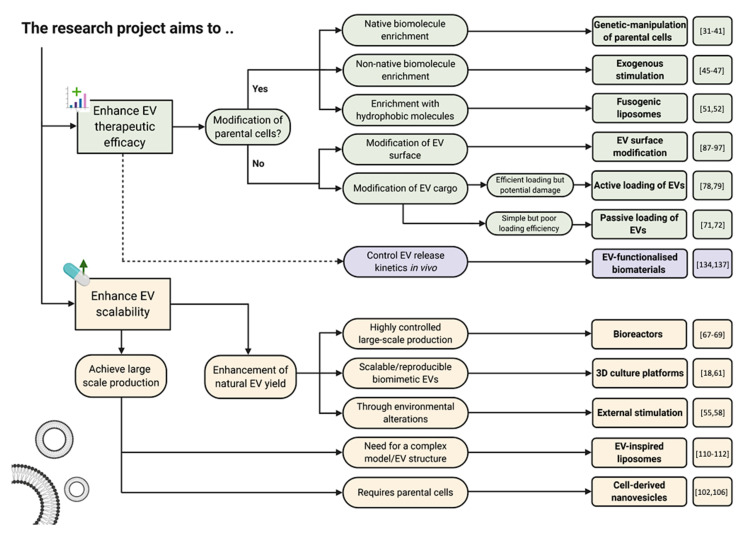
Schematic guide providing an overview of the engineering approaches and technologies currently used to enhance the therapeutic efficacy and scalability of EVs to improve their clinical utility.

In this review we highlighted the current state of the art EV-engineering techniques with discussion of opportunities and obstacles for each. This is synthesised into a guide for selecting a suitable strategy to maximise the potential efficacy of EVs as nanoscale therapeutics (Figure 9). When selecting an appropriate engineering approach, it is important to understand the intricacy of each strategy. For example, if the research conducted is looking to modify EVs therapeutic efficacy, this could be achieved via parental cell augmentation or direct modification of isolated EVs. If the route of parental cell modification is selected, several strategies have been utilised in the literature each with their own advantages/disadvantages and these will need to be explored early before settling on a specific experimental protocol.

## 6. Conclusions

Although great strides have been made in understanding the physiological utility of EVs, clinical translational has been limited by a number of formulation challenges. This has resulted in intensive research into methods to enhance EVs potency and yield. In this review, key areas, including the procurement of EVs from physiologically relevant environments, scalability of manufacturing, the fabrication of artificial nanoparticles and controlling in vivo release kinetics, have been identified as being crucial to the development of a clinically-viable EV product. With continued efforts, these strategies present an exciting platform to engineer EVs beyond their native function, thereby enhancing their potential clinical efficacy as acellular nanoscale therapeutics.

## Figures and Tables

**Table 1:** Methods to re-engineer the parental cell to modulate EVs.

Engineering Method	Advantages	Disadvantages	Ref.
Genetic-manipulation	Allows the enrichment of a certain therapeutic molecule of interest	Cumbersome and time-consuming	[31,32]
		High-associated costs	
		Issue regarding transduction and EV loading efficiency	
Exogenous stimulation	Avoids the issues associated with genetic reprogramming	Long compound exposure/concentration may affect cell viability	[45,46,47]
		EV loading efficiency	
Fusogenic liposomes	Delivery of both hydrophobic and hydrophilic cargo into the cell	Issues with loading large cargo	[50,51]
		Difficult to control proportion packaged into EVs	
External stimulation	Enhanced EVs yield/therapeutic potency	May alter composition of EVs produced	[55,56,57]
3D culture platforms	Enhanced EVs yield/therapeutic potency	Possible difficulty in extracting EVs	[18,61,64]
Bioreactors	Scalability/reproducibility of EV manufacture	Issues with the use of primary cells	[67,68,69]
		May alter composition of EVs	

**Table 2:** Post-isolation EV loading methods.

Technique	Advantages	Disadvantages	Ref.
Co-incubation	Simple method Membrane not compromised Inexpensive	Low loading efficacy	[71]
		Require hydrophobic cargo	
Electroporation	Allows loading of large molecules (siRNA, miRNA)	Disrupts EV integrity	[74]
		RNA aggregation	
Sonication	Higher loading efficiency	Possible membrane deformation	[78]
		Low-efficiency loading of hydrophobic cargo	
Extrusion	High loading efficiency	Possible membrane deformation	[79]
	Simple method		
Freeze/thaw	Medium loading efficiency	Aggregation of EVs	[83]
		Reduced loading efficacy	
Saponin-assisted loading	High loading efficiency	Possible membrane deformation	[80]
		Possible in vivo toxicity	

**Table 3:** EV surface modification methods.

Technique	Advantages	Disadvantages	Ref.
Hydrophobic insertion	Highly effective for the addition of lipophilic molecules	Dependent upon hydrophobic/amphiphilic properties	[87,88]
	Simple co-incubation		
Synthetic lipid nanoparticle fusion	Facilitate the transfer of membranes proteins	Risk of cargo leakage during fusion	[89,90]
	Allow more complex membrane modifications	More complex approach with the development of synthetic nanoparticles	
Covalent modifications	Rapid and simple	Possible alteration of the active site of surface proteins	[91,92]
	Good stability due to the strong covalent bond		
	Easily scalable		
Non-covalent modifications	High loading efficiency	Weaker bond strength compared to covalent link (less stable)	[95,96]
	Simple method	Potential membrane disruption when using cationic molecules	

**Table 4:** Delivery of EVs within biomaterial scaffolds for tissue regeneration in various models.

Clinical Application	EV Source	Biomaterial	Study Observations	Ref.
Cardiovascular	Endothelial progenitor cells	Adamantane and β-cyclodextrin-modified hyaluronic acid hydrogel	Enhanced myocardium regeneration	[126]
	Cardiomyocyte-derived iPSCs	Collagen type I Gelfoam sponge	Increased cardiac recovery in a rat myocardial infarction model	[127]
Skin	Blood plasma	Chitosan/silk fibroin sponge	Enhanced wound healing in a diabetic rat model	[128]
	Umbilical cord-derived MSCs	HydroMatrix hydrogel	Reduced myofibroblast accumulation/scar formation	[129]
Angiogenic	Human placental MSCs	Chitosan hydrogel	Improved EV retention and capillary formation in hindlimb ischemic model	[130]
Bone	BMSCs	HyStem-HP hydrogel	Increased bone healing of critical-sized calvaria defects	[131]
	MSCs	Thiol-modified hyaluronan	Accelerated regeneration of rat calvaria defect	[132]
Muscular	MSCs	Matrigel	Enhanced angiogenesis and muscle recovery in an ischemia model	[133]
	MSCs	Silk fibroin hydrogel	Prevented ischemia-induced vascular dysfunction	[116]
